# Contamination of clinical specimens with MLV-encoding nucleic acids: implications for XMRV and other candidate human retroviruses

**DOI:** 10.1186/1742-4690-7-112

**Published:** 2010-12-20

**Authors:** Robert A Smith

**Affiliations:** 1 Department of Pathology, University of Washington, Seattle WA, USA

## Abstract

Efforts to assess the prevalence of xenotropic murine leukemia virus-related virus (XMRV) in patients with prostate cancer and chronic fatigue syndrome have relied heavily on PCR-based testing of clinical samples and have yielded widely divergent findings. This week in *Retrovirology*, reports from four independent research groups illustrate the extreme care needed to exclude DNA or RNA contamination in PCR analyses of XMRV. In addition, phylogenetic evidence suggesting that previously-published XMRV sequences originated from a commonly-used prostate carcinoma cell line (22Rv1) is presented. These findings raise important questions regarding the provenance of XMRV and its potential connection to human disease.

## Introduction

Reports of a newly-discovered gammaretrovirus (xenotropic murine leukemia virus-related virus; XMRV) in patients diagnosed with prostate cancer [1,2] and chronic fatigue syndrome (CFS) [3] have attracted the attention of investigators throughout the retroviral research community. XMRV was initially identified in prostate tumor samples from individuals harboring a specific polymorphism in *RNASEL*, a gene important for interferon-mediated antiviral defense [1]. Studies describing the receptor usage and integration site preference of the virus were soon followed by a second report of XMRV infection in an unrelated cohort of prostate cancer patients [2]. Although an association between XMRV and the aforementioned *RNASEL *polymorphism was not found [2], the idea that defects in innate immunity might be linked to XMRV infection prompted others to look for the virus in patients with CFS [3]. Remarkably, PCR assays identified XMRV DNA in peripheral blood samples from 68 of 101 CFS patients and 8 of 218 healthy controls. These and other findings provided compelling evidence that XMRV is the first known example of an exogenous human gammaretrovirus.

In contrast, subsequent efforts to assess the prevalence of XMRV in patients with CFS and prostate cancer have reached widely disparate conclusions [[4]; see also reference [5] for review]. The underlying factors responsible for this discord are unclear; but from the beginning, researchers have repeatedly voiced concerns that at least some accounts of PCR-positive results are attributable to the inadvertent contamination of human specimens or reagents with mouse DNA. These concerns were revisited following a recent report by Lo *et al*. that described the existence of sequences closely related to polytropic and modified-polytropic murine leukemia viruses (MLVs)--but not XMRV--in blood samples from CFS patients [6]. Such skepticism is justified by previous examples of alleged human retroviruses that later turned out to be laboratory artifacts [7].

## Evidence for contamination of human samples

With this history in mind, four independent studies published this week in *Retrovirology *reinforce the need to take extreme precautions in excluding mouse DNA contamination. Robinson *et al. *[8] performed a PCR analysis of 437 prostate tissue specimens from patients in the United Kingdom (UK), Thailand and Korea using primers that targeted the 5'-leader region of XMRV *gag*. Initial PCR results showed that 14 of 292 samples from the UK contained XMRV or MLV-related sequences. However, 78 of the UK samples, including all 14 XMRV/MLV-positive specimens, contained amplifiable levels of LTR sequences from intercisternal A-type particles (IAPs), a class of endogenous retroelements found in the mouse genome. Similarly, Oakes and colleagues [9] identified 2 of 112 blood samples from CFS patients and 17 of 36 samples from healthy controls that were PCR-positive for XMRV *gag-*leader DNA, but later found that all of the XMRV-positive specimens contained amplifiable levels of mouse mitochondrial or IAP sequences. These data strongly suggest that the XMRV sequences recovered by Robinson [8] and Oakes [9] originated from mouse DNA that contaminated the study samples prior to PCR.

## Evidence for contamination of PCR reagents

A third report by Sato *et al. *[10] describes the detection of MLV-encoding nucleic acids in PCR reagents obtained from a commercial supplier (Invitrogen). Analyses of individual components from the PCR kit (SuperScript^® ^III One-Step RT-PCR System with Platinum^® ^Taq High Fidelity) suggest that the mixture of reverse transcriptase and Taq DNA polymerase supplied by the manufacturer was contaminated with MLV RNA. This contamination likely originated from a monoclonal antibody preparation used in the polymerase mixture to facilitate hot-start PCR.

Further details of the contamination found by Robinson [8], Oakes [9] and Sato [10] were obtained by DNA sequence analysis of the PCR-amplified products. All three studies identified sequences that that were closely related to endogenous MLV. In particular, Oakes and coworkers obtained a broad array of polytropic, modified-polytropic and xenotropic MLV-like sequences [9], a result strikingly similar to the findings of Lo *et al*. in their analysis of CFS patient samples [6]. Both Oakes and Robinson also identified subsets of amplicons that encoded a 24-nt *gag*-leader deletion previously thought to be specific for XMRV (see below). Collectively, these data show the ease with which contamination can lead to false-positive MLV/XMRV signals.

## Phylogenetic support for contamination in previous studies of XMRV

Finally, Hué and colleagues [11] present multiple lines of evidence suggesting that contamination has occurred repeatedly in previous studies of XMRV. The authors begin by showing that the 24-nt *gag*-leader deletion is not unique to XMRV; PCR primers targeting the deletion readily amplified endogenous MLV sequences from 12 different inbred and wild-derived mouse strains commonly used in laboratory experiments, as well as MLV sequences present in 5 of 411 human tumor cell lines. The latter result is consistent with previous reports of xenotropic MLV contamination in human cell cultures [[11] and references therein].

Next, Hué *et al*. PCR-amplified, cloned and sequenced XMRV *gag*, *pol *and *env *segments from 22Rv1 prostate carcinoma cells, an immortalized line known to harbor multiple integrated copies of the virus. Remarkably, the 22Rv1 sequences displayed average pairwise genetic distances that equaled or exceeded those of previously-published XMRV sequences from prostate cancer [1] and CSF patients [3], despite the fact that these patients were from epidemiologically unlinked cohorts. In addition, phylogenetic analyses of the 22Rv1 and patient-derived XMRV sequences strongly suggest that the patient sequences obtained to date [1,3] originated from one or more XMRV proviruses present in the 22Rv1 cell line [11].

## Conclusions

The reports discussed above [8-11] collectively identify three potential sources of contamination in PCR-based studies of XMRV: (i) MLV-encoding nucleic acids present in commercial PCR reagents, (ii) trace amounts of mouse genomic DNA in human blood and tissue samples, and (iii) DNA or RNA from human tumor cell lines infected with XMRV or other closely-related gammaretroviruses. PCR testing for IAP sequences [8,9] should prove useful in further studies of XMRV, as well as other candidate human retroviruses, in which the confounding effects of mouse DNA contamination must be minimized. However, the findings of Hué *et al*. clearly show that contamination cannot be assessed by PCR testing for mouse DNA alone, since several human cell lines harbor xenotropic MLVs that are closely related to XMRV [11]. Additional findings from the Hué study suggest that previously-published XMRV sequences [1,3] were derived from copies of the virus present in 22Rv1 cells, which likely acquired XMRV during xenografting of the tumor cells in athymic mice [[11] and references therein]. Collectively, these results cast serious doubts on the PCR evidence used to support claims of MLV-related viruses in prostate cancer and CFS patients. Future assessments of the prevalence of XMRV should include more rigorous PCR and phylogenetic tests to exclude the possibility of contamination.

## Abbreviations

XMRV: xenotropic murine leukemia virus-related virus; MLV: murine leukemia virus; PCR: polymerase chain reaction; CFS: chronic fatigue syndrome; IAP: intercisternal A-type particle

## Competing interests

The author declares that he has no competing interests.

## References

[B1] Urisman A Molinaro RJ Fischer N Plummer SJ Casey G Klein EA Malathi K Magi-Galluzzi C Tubbs RR Ganem D Silverman RH DeRisi JL Identification of a novel gammaretrovirus in prostate tumors of patients homozygous for R462Q RNASEL variant PLoS Pathog 2006 2 e25 10.1371/journal.ppat.0020025 16609730 PMC1434790

[B2] Schlaberg R Choe DJ Brown KR Thaker HM Singh IR XMRV is present in malignant prostatic epithelium and is associated with prostate cancer, especially high-grade tumors Proc Natl Acad Sci USA 2009 106 16351 16356 10.1073/pnas.0906922106 19805305 PMC2739868

[B3] Lombardi VC Ruscetti FW Das Gupta J Pfost MA Hagen KS Peterson DL Ruscetti SK Bagni RK Petrow-Sadowski C Gold B Dean M Silverman RH Mikovits JA Detection of an Infectious Retrovirus, XMRV, in Blood Cells of Patients with Chronic Fatigue Syndrome Science 2009 326 585 589 10.1126/science.1179052 19815723

[B4] Hohn O Krause H Barbarotto P Niederstadt L Beimforde N Denner J Miller K Kurth R Bannert N Lack of evidence for xenotropic murine leukemia virus-related virus (XMRV) in German prostate cancer patients Retrovirology 2009 6 92 10.1186/1742-4690-6-92 19835577 PMC2770519

[B5] Silverman RH Nguyen C Weight CJ Klein EA The human retrovirus XMRV in prostate cancer and chronic fatigue syndrome Nat Rev Urol 2010 7 392 402 10.1038/nrurol.2010.77 20517289

[B6] Lo S-C Pripuzova N Li B Komaroff AL Hung G-C Wang R Alter HJ Detection of MLV-related virus gene sequences in blood of patients with chronic fatigue syndrome and healthy blood donors Proc Natl Acad Sci 2010 107 15874 15879 10.1073/pnas.1006901107 20798047 PMC2936598

[B7] Weiss RA A cautionary tale of virus and disease BMC Biol 2010 8 124 10.1186/1741-7007-8-124 20920148 PMC2946284

[B8] Robinson M Erlwein O Kaye S Weber J Cingoz O Patel A Walker M Kim W-J Uiprasertkul M Coffin JM McClure MO Mouse DNA contamination in human tissue tested for XMRV Retrovirology 2010 7 108 21171966 10.1186/1742-4690-7-108 PMC3019155

[B9] Oakes B Tai AK Cingoz O Henefield MH Levine S Coffin JM Huber BT Contamination of clinical DNA samples with mouse DNA can lead to false detection of XMRV-like sequences Retrovirology 2010 7 109 21171973 10.1186/1742-4690-7-109 PMC3022687

[B10] Sato E Furuta RA Miyazawa T An endogenous murine leukemia viral genome contaminant in a commercial RT-PCR kit is amplified using standard primers for XMRV Retrovirology 2010 7 110 10.1186/1742-4690-7-110 21171978 PMC3024226

[B11] Hué S Gray ER Gall A Katzourakis A Tan CP Houldcroft CJ McLaren S Pillay D Futreal A Garson JA Pybus OG Kellam P Towers GJ Disease-associated XMRV sequences are consistent with laboratory contamination Retrovirology 2010 7 111 21171979 10.1186/1742-4690-7-111 PMC3018392

